# Classical risk factors, but not HPV status, predict survival after chemoradiotherapy in advanced head and neck cancer patients

**DOI:** 10.1007/s00432-016-2203-7

**Published:** 2016-07-01

**Authors:** Géraldine Descamps, Yasemin Karaca, Jérôme R Lechien, Nadège Kindt, Christine Decaestecker, Myriam Remmelink, Denis Larsimont, Guy Andry, Samantha Hassid, Alexandra Rodriguez, Mohammad Khalife, Fabrice Journe, Sven Saussez

**Affiliations:** 1 Laboratory of Anatomy and Cell Biology, Faculty of Medicine and Pharmacy, University of Mons, Pentagone 2A, Avenue du Champ de Mars, 6, 7000 Mons, Belgium; 2 Laboratories of Image, Signal Processing and Acoustics, Ecole polytechnique de Bruxelles, Université Libre de Bruxelles (ULB), Brussels, Belgium; 3 Department of Pathology, Hôpital Erasme, Université Libre de Bruxelles (ULB), Brussels, Belgium; 4 Department of Pathology, Bordet Institute, Université Libre de Bruxelles (ULB), Brussels, Belgium; 5 Department of Head and Neck Surgery, Bordet Institute, Université Libre de Bruxelles (ULB), Brussels, Belgium; 6 Department of Oto-Rhino-Laryngology, CHU Saint-Pierre, Université Libre de Bruxelles (ULB), Brussels, Belgium; 7 Department of Head and Neck Surgery, EpiCURA, Baudour, Belgium; 8 Laboratory of Oncology and Experimental Surgery, Bordet Institute, Université Libre de Bruxelles (ULB), Brussels, Belgium

**Keywords:** Head and neck cancers, HPV, Tobacco, Alcohol, Survival, Concomitant chemoradiotherapy

## Abstract

**Purpose:**

Despite the advent of concomitant chemoradiotherapy (CCRT), the prognosis of advanced head and neck squamous cell carcinoma (HNSCC) patients remains particularly poor. Classically, HNSCC, especially oropharyngeal carcinomas, associated with human papillomavirus (HPV) exhibits better treatment outcomes than HNSCCs in non-infected patients, eliciting a call for the de-escalation of current therapies. To improve the management of HNSCC patients, we aimed to determine the impact of active HPV infection on patient response, recurrence and survival after CCRT in a population of heavy tobacco and alcohol consumers.

**Methods:**

Paraffin-embedded samples from 218 advanced HNSCC patients, mostly smokers and/or drinkers treated by CCRT, were tested for the presence of HPV DNA by surrogate type-specific E6/E7 qPCR and p16 immunohistochemistry. Associations between the response to CCRT and patient outcomes according to HPV status and clinical data were evaluated by Kaplan–Meier analysis and both univariate and multivariate Cox regression.

**Results:**

Type-specific E6/E7 PCR demonstrated HPV positivity in 20 % of HNSCC. Regarding HPV status, we did not find any significant relation with response to therapy in terms of progression-free survival or overall survival. However, we observed a significantly worse prognosis for consumers of alcohol and tobacco compared to nondrinkers (*p* = 0.003) and non-smokers (*p* = 0.03). Survival analyses also revealed that the outcome is compromised in stage IV patients (*p* = 0.007) and, in particular, for oral cavity, hypopharynx and oropharynx carcinoma patients (*p* = 0.001).

**Conclusion:**

The risk of death from HNSCC significantly increases when patients are exposed to tobacco and alcohol during their therapy, regardless of HPV status.

## Introduction

Head and neck squamous cell carcinoma (HNSCC) represents the fifth most common malignancy diagnosed worldwide. In 2012, HNSCCs accounted for approximately 59.000 new cases in the USA and more than 77.000 in Western and Eastern Europe (Globocan, 2012). These cancers form a group of heterogeneous tumors presenting distinct etiology, histology, risk factors and treatment approaches. Over the last twenty years, a clear increase in the incidence of oropharyngeal and oral cavity carcinomas has been observed, particularly in young adults in both the US and Europe, whereas the incidence of laryngeal carcinomas tended to remain stable or decrease slightly (D’Souza et al. 2007; Sturgis and Cinciripini 2007).

The advent of concomitant chemoradiotherapy (CCRT) in the early 2000s occurred in the phase III trial of Forastière et al’s study (Forastiere et al. 2003), who reported that the use of high-dose cisplatin and radiotherapy resulted in a considerable improvement in the survival of patients with laryngeal cancer. In addition, reduced mortality and improved locoregional control were observed upon treatment with both cetuximab and radiotherapy (Bonner et al. 2006). Currently, CCRT remains the gold standard to treat primary locally advanced head and neck cancer patients, especially those with stage III and IV disease (Forastiere et al. 2003; Bonner et al. 2006; Rosenthal et al. 2015). However, these aggressive treatments are characterized by tissue sequela (i.e., dry mucosa, muscle atrophy, and fibrosis leading to acute and chronic toxicities), morbidity (10 % of tracheotomy cases), and mortality (Trotti et al. 2003; Lazarus 2009; Hu et al. 2012; Hutcheson and Lewin 2012). It is therefore crucial to predict which patients will benefit from CCRT by investigating the impact of risk factors on the response to treatment.

Patients with HNSCCs often present a long history of tobacco and alcohol use. Recently, human papillomavirus (HPV) infection has emerged as an additional risk factor and could be involved in increased worldwide incidence of a subset of HNSCCs, especially oropharyngeal cancers (Fakhry and Gillison 2006; Sturgis and Cinciripini 2007; Fakhry et al. 2014). The development of cancers related to HPV infections has significantly complicated the profile of head and neck cancer patients, notably in terms of prognosis and response to treatment. The management of such patients is particularly complex in Europe, where many individuals are heavy smokers and/or drinkers (Duray et al. 2012; Duray et al. 2013). Indeed, while non-smoking and nondrinking oropharyngeal patients exhibit an improved response to therapy and a better outcome, tobacco and alcohol consumers with non-oropharyngeal cancers are associated with a heterogeneous prognosis (Ang et al. 2010; Isayeva et al. 2012). In this context, controversy exists regarding the prognosis of HPV+ patients treated by CCRT. Whereas several studies have reported that HPV infection is associated with a good prognosis (Kumar et al. 2007; Fakhry et al. 2008; Ang et al. 2010; Rischin et al. 2010; Hong et al. 2010; Nygård et al. 2012), other groups have reported opposing findings (Rosenquist et al. 2007; Lee et al. 2012; Duray et al. 2012). Thus, studies investigating the HPV status of HNSCC patients must be interpreted with caution because many are small clinical series without information regarding the alcohol consumption and smoking status of the patients.

The present study aims to determine the influence of HPV status on the response to CCRT and to estimate the impact of HPV infection as well as tobacco and alcohol consumption on recurrence and survival in a retrospective and prospective analysis of 218 head and neck cancer patients.

## Materials and methods

### Study population and clinical data

Formalin-fixed, paraffin-embedded HNSCC specimens were obtained from 218 patients (173 males, 45 females) who underwent concomitant chemoradiotherapy at the Saint-Pieter Hospital (Brussels) and Epicura Hospital (Baudour). Patients treated by cisplatin or Erbitux concomitant with radiotherapy were included in this study, and more than 95 % of the patients were stage III or IV. The response to treatment was evaluated three months after the end of treatment based on a clinical examination (endoscopy) and imaging technique (CT scan or MRI). On the basis of their cigarette and alcohol exposure, participants were classified as current, former or non-smokers and nondrinkers. Smokers/drinkers were defined as patients who continue to smoke and/or drink during their treatment, while formers include patients who stopped their consumption at diagnosis or for years before. Non-smokers and nondrinkers are individuals who have never used tobacco or alcohol. The clinical data collected from this series of 218 HNSCC patients are detailed in Table 1. This prospective and retrospective study was approved by the Institutional Review Board (AK/09-09-47/3805, P2014/185, as/2319).

**Table 1:** Clinical data of the whole population and regarding HPV status

Variables	All patients *n* = 218 (%)	HPV−^a^ *n* = 170	HPV+/p16− *n* = 26	HPV+/p16+ *n* = 17	*p* value^b^
Sex					0.3
Male	173 (80 %)	138 (81 %)	20 (77 %)	11 (65 %)	
Female	45 (20 %)	32 (19 %)	6 (23 %)	6 (35 %)	
Age (years)					
Range	21–88	21–88	43–83	48–79	
Mean	59	59	57	63	
Median	58	58	54	60	
Localization					0.007
Oral cavity	38 (17 %)	34 (20 %)	3 (12 %)	1 (6 %)	
Oropharynx	78 (36 %)	55 (32 %)	10 (38 %)	12 (70 %)	
Hypopharynx	46 (21 %)	39 (23 %)	5 (19 %)	0 (0 %)	
Larynx	37 (17 %)	26 (15 %)	8 (31 %)	1 (6 %)	
Nasopharynx	19 (9 %)	16 (10 %)	0 (0 %)	3 (18 %)	
TNM stage					0.7
I	0 (0 %)	0 (0 %)	0 (0 %)	0 (0 %)	
II	4 (2 %)	3 (2 %)	1 (4 %)	0 (0 %)	
III	49 (22 %)	41 (24 %)	4 (15 %)	3 (18 %)	
IV	161 (74 %)	122 (72 %)	21 (81 %)	14 (82 %)	
Not recorded	4 (2 %)	4 (2 %)	0 (0 %)	0 (0 %)	
Risk factors					
Tobacco					0.9
Smoker	130 (59.5 %)	100 (59 %)	16 (62 %)	10 (59 %)	
Non-smoker	24 (11 %)	18 (10.5 %)	3 (11 %)	3 (18 %)	
Former smoker	63 (29 %)	51(30 %)	7 (27 %)	4 (23 %)	
Not recorded	1 (0.5 %)	1 (0.5 %)	0 (0 %)	0 (0 %)	
Alcohol					0.3
Drinker	130 (59.5 %)	99 (58 %)	18 (69 %)	8 (47 %)	
Nondrinker	50 (23 %)	38 (22.5 %)	5 (19 %)	7 (41 %)	
Former drinker	37 (17 %)	32 (19 %)	3 (12 %)	2 (12 %)	
Not recorded	1 (0.5 %)	1 (0.5 %)	0 (0 %)	0 (0 %)	
Treatment					0.2
*Cisplatin/carboplatin	173 (79.5 %)	137 (80.5 %)	20 (77 %)	12 (70 %)	
*Erbitux	34 (15.5 %)	22 (13 %)	6 (23 %)	5 (30 %)	
Not recorded	11 (5 %)	11 (6.5 %)	0 (0 %)	0 (0 %)	
Treatment details					
*Cisplatin					
100 mg/m^2^ (day 1: 21–42)	60				
One dose	1				
Two doses	15				
Three doses	35				
> Three doses	9				
*Cisplatin					
40 mg/m^2^ weekly	18				
*Cisplatin > 100 mg/m^2^	2				
*Erbitux	34				
*Cisplatin + 5FU	19				
*Cisplatin +					
Carboplatin	3				
*Carboplatin	11				
*Not recorded	11				
Radiotherapy (GY)					
70	103 (47 %)				
>70	81 (37 %)				
<70	9 (4 %)				
Not recorded	25 (12 %)				
Responders					0.7
Yes	116 (53 %)	93 (55 %)	12 (46 %)	9 (53 %)	
No	102 (47 %)	77 (45 %)	14 (54 %)	8 (47 %)	
Recurrence^c^					
Yes	90 (41 %)	72 (42 %)	10 (38 %)	6 (35 %)	0.6
No	128 (59 %)	98 (58 %)	16 (62 %)	11 (65 %)	
Progression-free survival (PFS) (months)					
Range	1–144	1–144	2–105	2–71	
Mean	28	30	22	24	
Median	14	14	11	19	
Overall survival (OS)					
Range	1–180	1–180	2–105	2–72	
Mean	36	38	27	26	
Median	24	24	17	19	
Follow-up (months)					
Range	1–180	1–180	2–105	2–72	
Mean	36	36	28	27	
Median	24	24	17	19	

^a^ *HPV* human papillomavirus, *GY* Gray, *HNSCC* head and neck squamous cell carcinoma

^b^Pearson’s Chi-square test comparing HPV−, HPV+/p16− and HPV+/p16+ groups

^c^Recurrence include local and/or nodal and/or distant metastases recurrences

### DNA extraction and real-time PCR amplification of HPV type-specific DNA

The formalin-fixed, paraffin-embedded tissue samples (*n* = 218) were sectioned (10 × 5 µm), deparaffinized, and digested with proteinase K by overnight incubation at 56 °C. DNA was purified using the QIAamp DNA Mini Kit (Qiagen, Benelux, Belgium) according to the manufacturer’s recommended protocol. All DNA extracts were tested for the presence of 18 different HPV genotypes using TaqMan-based real-time PCR, as described previously (Depuydt et al. 2006, 2007; Duray et al. 2013).

### p16 immunohistochemistry

Each HPV-positive case was further immunohistochemically evaluated for p16 expression using the recommended mouse monoclonal antibody (CINtec p16, Ventana, Tucson, USA) (Sawicka et al. 2013) and an automated immunostaining protocol (Bond-Max, Leica Microsystems, Wetzlar, Germany). Immunohistochemistry was performed on 5-µm thick tissue sections in the Leica Bond-Max immunostainer: The sections were deparaffinized, submerged in epitope retrieval solution (pH 6) for 10 min, and incubated with CINtec p16 antibody for 30 min. Then, polymer detection was performed using Bond Polymer Refine Detection according to the manufacturer’s protocol (Leica, Wetzlar, Germany), and the slides were counterstained with hematoxylin and luxol fast blue. Tissue sections from cervix lesions were used as positive controls. p16 expression was deemed positive only when the staining was both nuclear and cytoplasmic and when over 70 % of tumor cells were stained (Smeets et al. 2007).

### Statistical analysis

Independent groups of categorical data were compared using the Pearson Chi-square test. Progression-free survival (PFS) and overall survival (OS) data were measured in terms of months from the date of diagnosis until disease recurrence or death or until the date at which the patient was last known to be alive. Standard survival time analyses were performed using Kaplan–Meier curves. For comparing two (or more) curves, univariate analyses were performed using the Cox regression model to estimate hazard ratios (HR), 95 % confidence intervals (CI), and associated *p* values. *p* values <0.05 were considered statistically significant. Multivariate Cox regression models were used to analyze the independent contribution of the HPV status to survival time in presence of other covariates such as conventional risk factors (stage, tobacco, alcohol) and response to CCRT. All statistical analyses were performed using Statistica (Statsoft, Tusla, OK, USA) and SPSS 15.0 Inc. (Chicago, IL, USA).

## Results

### Clinical data related to response, recurrence and survival in HNSCC patients

**Fig. 1:**
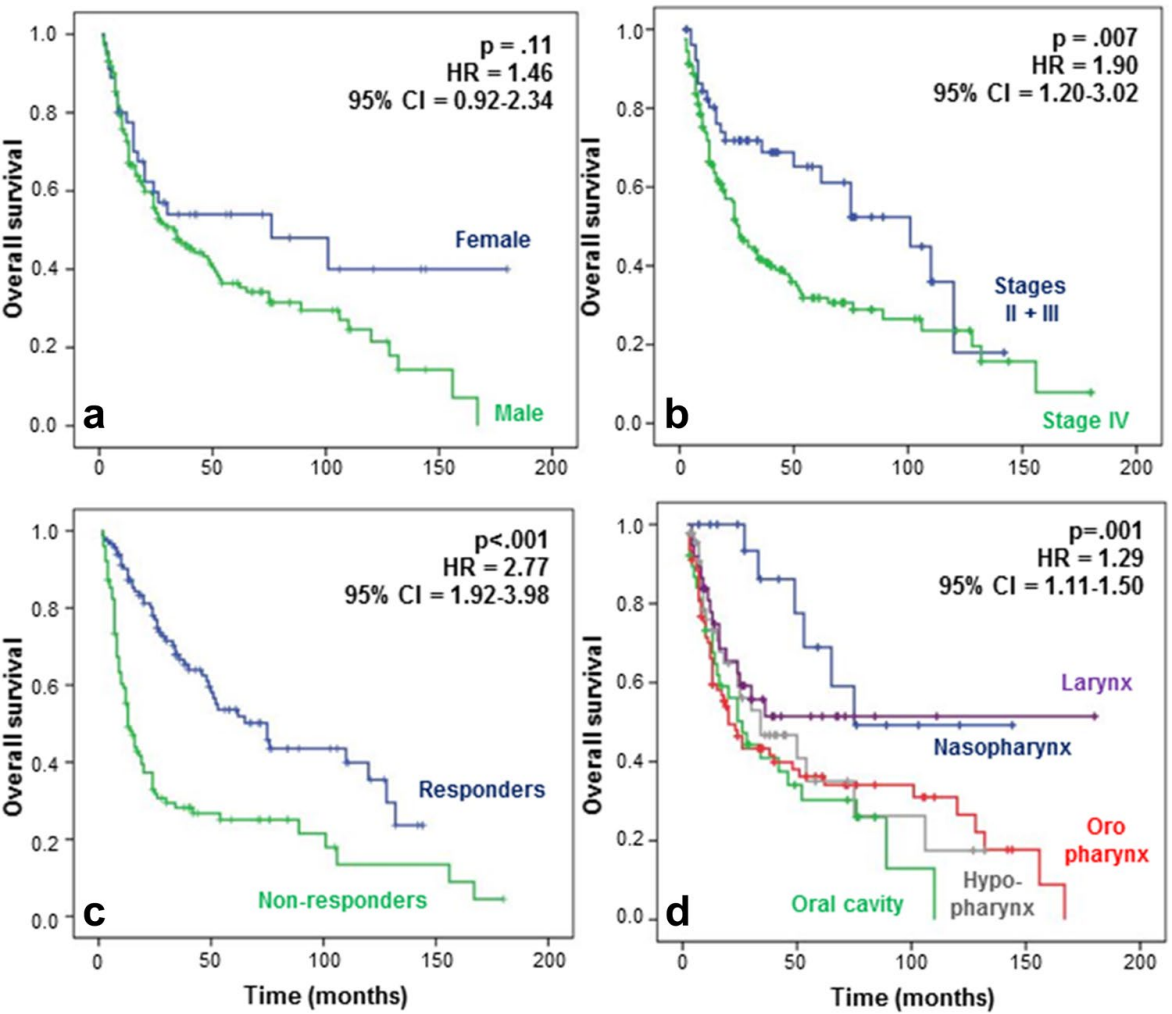
Overall survival observed by **a** gender, **b** stages, **c** response rate to CCRT, and **d** tumor location for patients with HNSCCs. Prognosis appeared to be better for women than men, but the difference was not significant (hazard ratio [HR], 1.46; 95 % CI 0.92–2.34; *p* 0.11). Patients with stages II and III tumors had a significantly longer OS than patients with stage IV tumors (HR 1.90; 95 % CI 1.20–3.02; *p* 0.007), and non-responders to CCRT had a shorter lifetime than responders (HR 2.77; 95 % CI 1.92–3.98; *p* < 0.001). Regarding tumor location, the OS was significantly better for the patients with laryngeal and nasopharyngeal tumors compared to the patients with oral cavity, hypopharyngeal and oropharyngeal tumors (HR 1.29; 95 % CI 1.11–1.50; *p* 0.001)

The response to CCRT, recurrence, and survival has been correlated with the different clinical data (Table 1). In terms of response and recurrence, only nasopharyngeal carcinoma fared significantly better than other cancers (*p* = 0.003) (data not shown). Gender did not significantly impact survival, although women seemed to present a higher lifetime survival (*p* = 0.11) (Fig. 1a). As expected, stage II and III patients had a longer survival compared to stage IV patients (*p* = 0.007) (Fig. 1b), and survival was also significantly higher for patients who responded to CCRT compared to non-responders (*p* < 0.001) (Fig. 1c). We also demonstrated that patients with nasopharyngeal or laryngeal carcinomas had a significantly better outcome than patients with carcinoma of the oropharynx, hypopharynx, or oral cavity (*p* = 0.001) (Fig. 1d).

### HPV status and relation with clinical data in HNSCC patients

**Fig. 2:**
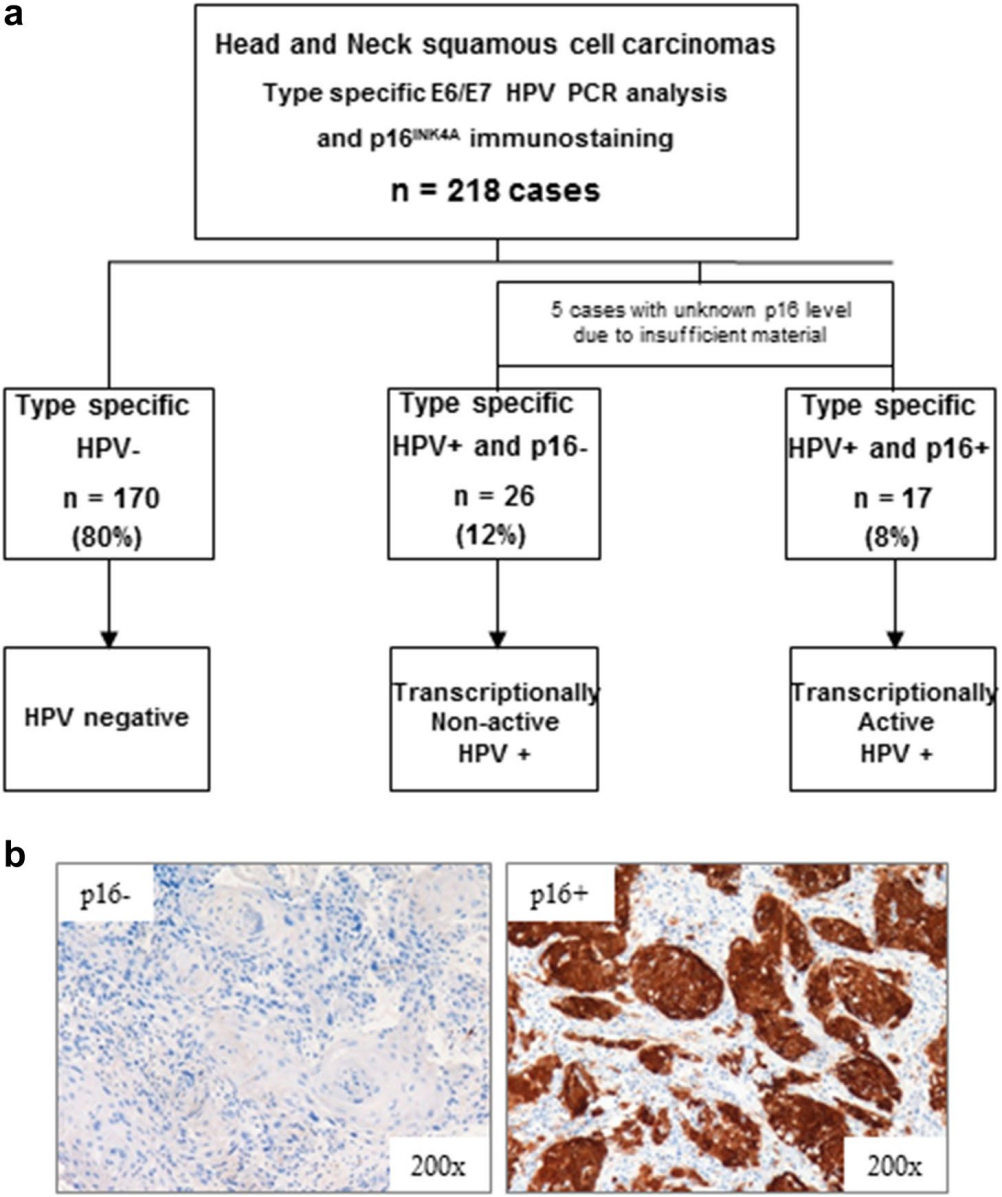
Assessment of HPV status in HNSCC patients. **a** HPV evaluation from 218 tumor tissues determined by real-time PCR and p16 immunohistochemistry. Five samples could not be analyzed by immunohistochemistry due to insufficient material. Among the 213 remaining cases, 170 (80 %) were negative for HPV, 26 (12 %) were positive for HPV but negative for p16, and 17 (8 %) were positive for both infection and p16 expression. **b** Typical p16 immunohistochemical expression corresponding to a transcriptionally active infection (p16+) and a non-active infection where p16 is not expressed (p16−)

The 218 patients treated by CCRT were genotyped via real-time PCR using primers for 18 different HPV types (Fig. 2). HPV-positive cases were next analyzed for p16 immunohistochemical expression to distinguish transcriptionally active infections (p16+) from non-active infections (p16−) (Fig. 2b). Among our 218 patients, we identified 17 patients (8 %) whose tumors were positive for high-risk HPV and for p16, whereas 26 patients (12 %) infected by HPV were p16-negative, corresponding to a latent HPV infection. Among the HPV+ population, 5 cases presented insufficient tissue quantity for p16 immunohistochemistry, and therefore, they were excluded from the analyses. Overall, 170 patients (80 %) presented HPV- tumors according to real-time PCR analysis (Fig. 2a).

The HPV+/p16+ group was composed of more men than women ranging from 48–79 years of age and mostly presenting stage IV disease (3 stage III and 14 stage IV) (Table 1). HPV+/p16+ cancers are more likely to develop in the oropharynx compared to other localizations (*p* = 0.007). In this subgroup of patients, there was a clear predominance of smokers (*n* = 10, 59 %) compared to patients who are former smokers (*n* = 4, 23 %) or who did not consume tobacco (*n* = 3, 18 %). In addition, about half of the patients were drinkers (*n* = 8, 47 %), but the proportion of nondrinkers was also high (*n* = 7, 41 %). However, no significant relation was found between HPV status and clinical data, including gender, smoking status, alcohol status, TNM stage, and treatment (Table 1).

### Relation between HPV status and response rate to CCRT in HNSCC patients

Using the Pearson’s Chi-square test, we investigated whether HPV positivity is related to the rate of response to CCRT. For the three groups of patients (HPV−, HPV+/p16− and HPV+/p16+), there was no significant difference in the rate of responders versus non-responders because the percentage of responders versus non-responders was only slightly higher in HPV− (55 vs. 45 %) and HPV+/p16+ patients (53 vs. 47 %) (*p* = 0.7) (Table 1).

### Relation between HPV infection, recurrence and survival in HNSCC patients

**Fig. 3:**
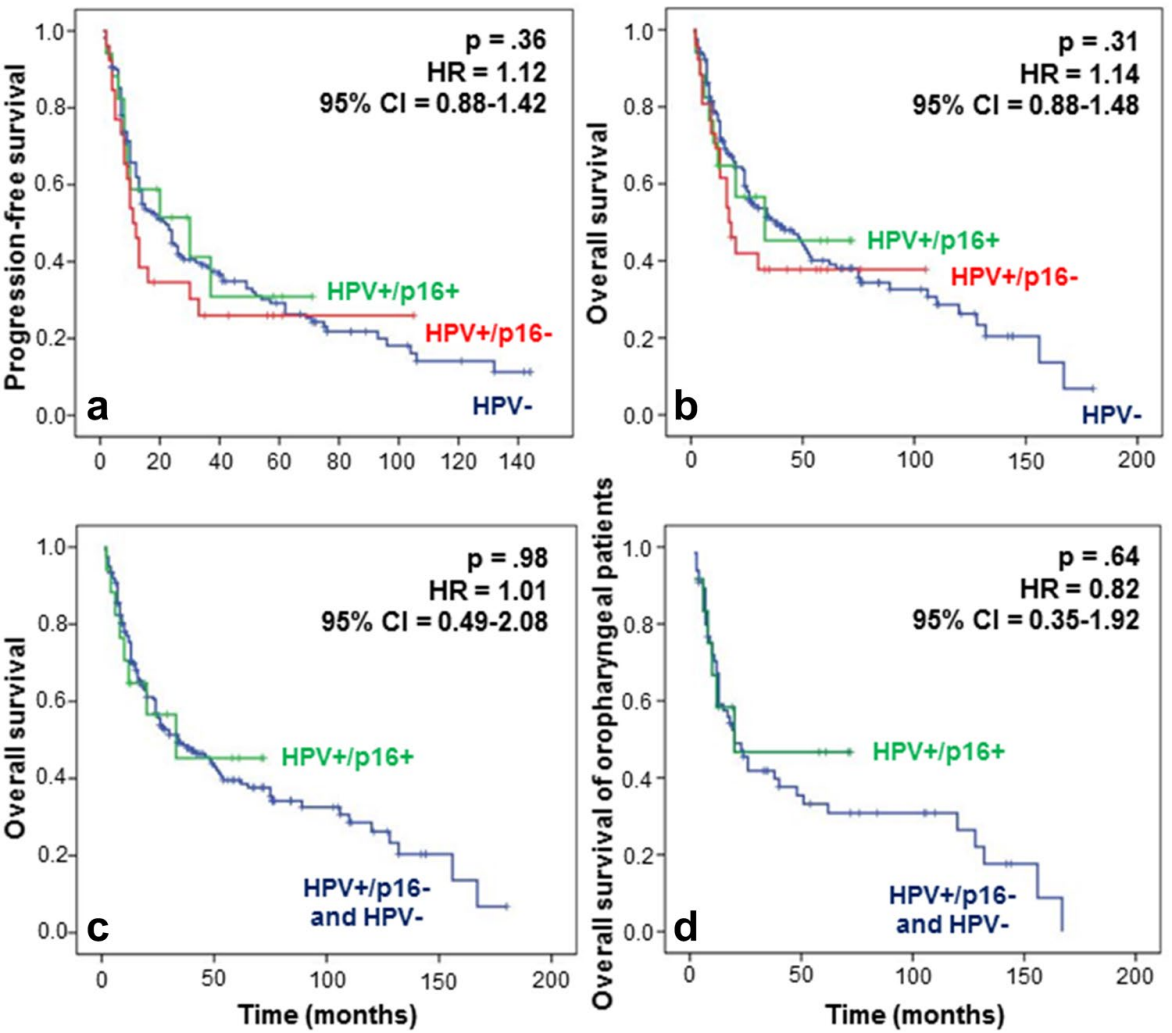
Evaluation of **a** PFS and **b** OS regarding HPV infection for patients with HNSCCs. Patients with HPV+/p16+ or HPV+/p16− tumors do not have a significant longer PFS (HR 1.12; 95 % CI 0.88–1.42; *p* 0.36) or OS (HR 1.14; 95 % CI 0.88–1.48; *p* 0.31) compared to HPV− patients. **c** Grouping patients according to transcriptionally active and non-active infection reveals no significant difference in OS (HR 1.01; 95 % CI 0.49–2.08; *p* 0.98) between the HPV+ group and the HPV− and HPV+/p16− groups. **d** Regarding the patients with oropharyngeal tumors, a trend to a better outcome was noted for the HPV+/p16+ patients, but the difference was not significant (HR 0.82; 95 % CI 0.35–1.92; *p* 0.64)

We did not observe any significant difference between the three populations of patients grouped by HPV status in terms of recurrence and survival (Fig. 3a, b). Indeed, at 5 years, the overall survival (OS) was slightly superior in the HPV+/p16+ subgroup, with 46 % of patients versus 38 and 40 % for both the HPV+/p16− and HPV− subgroups, respectively (Fig. 3b). However, this difference was not statistically significant (*p* > 0.05). Regarding Fig. 3a, the progression-free survival (PFS) at 5 years seemed to be slightly better for patients in the HPV+/p16+ subgroup compared to HPV+/p16− and HPV− patients, although the difference was not significant. We also evaluated the impact of a transcriptionally active infection on OS and compared the HPV+/p16+ patient group (active HPV) to a group combining HPV− and HPV+/p16− patients without finding any difference (Fig. 3c). PFS and OS were also evaluated in oropharyngeal carcinoma patients with respect to HPV status. The risk of death did not differ significantly between the HPV− and HPV+ patient groups, although we noted a trend to a better OS in active HPV patients, with a survival of 48 % at 5 years versus 31 % in the HPV− group (Fig. 3d), emphasizing the need to increase the HPV+/p16+ cohort to increase the statistical power. Moreover, we assessed the impact of HPV positivity on response and non-response to CCRT in patients affected by an oropharyngeal cancer, but we failed to demonstrate a significant relation between HPV infection and treatment response (Pearson’s Chi-square test, *p* = 0.4).

### Smoking/drinking habits and HPV infection related to survival in HNSCC patients

**Fig. 4:**
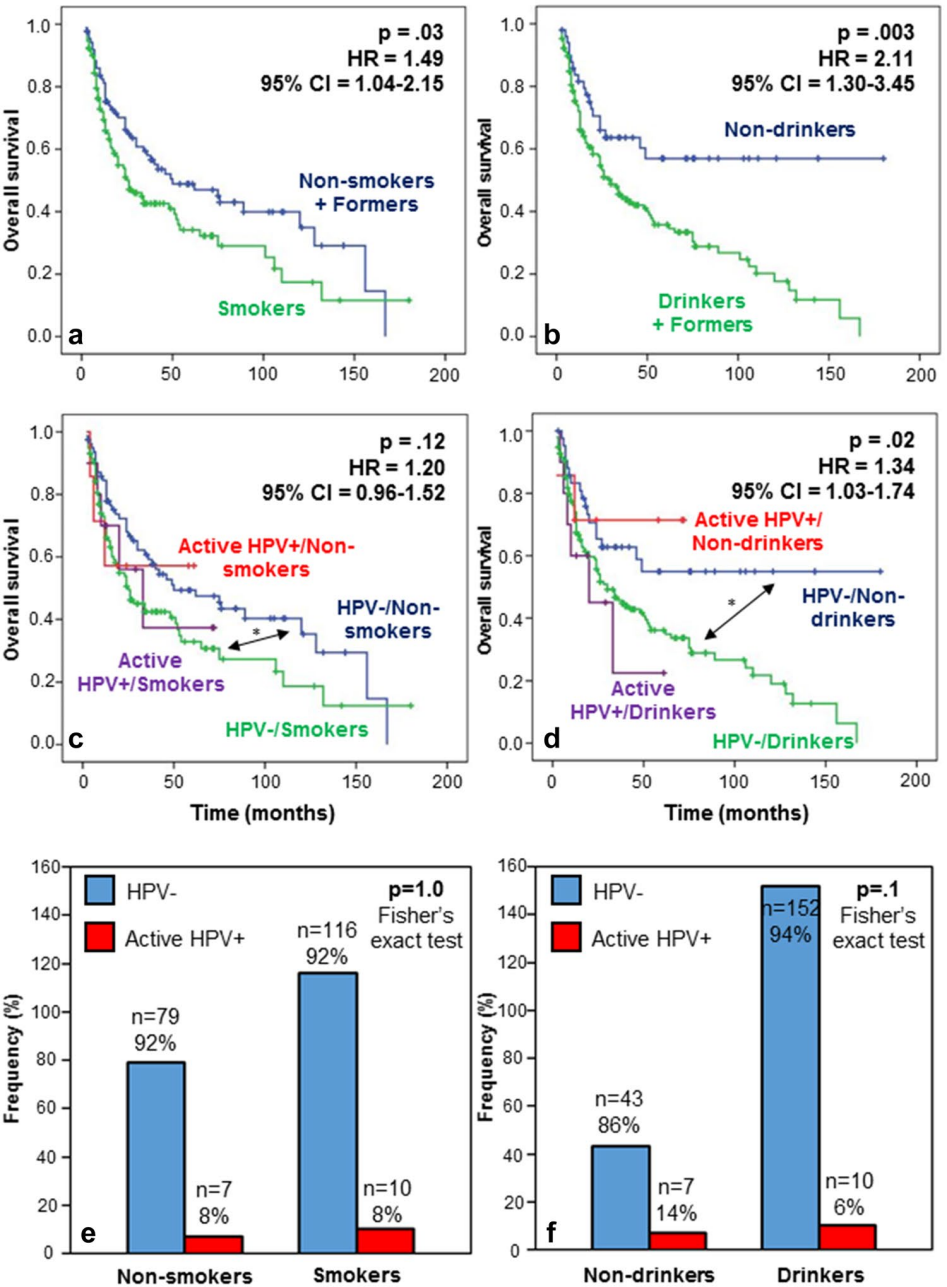
Survival and tobacco/alcohol habits with respect to HPV status in HNSCC patients. **a** OS by smoking status. The prognosis for non- and former smokers was significantly better than for smokers (HR 1.49; 95 % CI 1.04–2.15; *p* 0.03). **b** OS by drinking status. The prognosis for nondrinkers was significantly better than for former and current drinkers (HR 2.11; 95 % CI 1.30–3.45; *p* 0.003). **c** Patient OS regarding HPV and smoking status. HPV status did not affect prognosis. *The HPV− non-smokers had a significantly longer OS than the HPV− smokers (HR 1.55; 95 % CI 1.06–2.27; *p* 0.025). **d** OS regarding HPV and drinking status. The analysis revealed a significant difference between the four survival curves (HR 1.34; 95 % CI 1.03–1.74; *p* 0.02). Moreover, *the HPV− nondrinkers had a greater chance of survival compared to the HPV− drinkers (HR 2.04; 95 % CI 1.22–3.43; *p* 0.007). **e** Fisher’s exact test illustrating the frequency of HPV+ and HPV− patients among smoker and non-smoker patients. No difference was observed in the proportion of active HPV+ patients among non-smokers or smokers (8 versus 8 % *p* = 1.0). **f** Fisher’s exact test illustrating the frequency of HPV+ and HPV− patients among drinker and nondrinker patients. The frequency of HPV+ patients was slightly higher among nondrinkers without statistical significance (14 versus 6 %, *p* 0.1)

Considering the high prevalence of smokers/former smokers and drinkers/former drinkers in our population, we evaluated whether the clinical patient outcome is compromised by smoking/drinking habits regardless of HPV status. We analyzed the impact on survival for non-smokers, former smokers and smokers separately and did not observe any significant differences. However, we also compared a group of non-smokers and former smokers against a group of smokers (who represent a large majority) and observed a significant association between smoking and a worse prognosis (*p* = 0.03) (Fig. 4a). Consistent with this finding, we similarly analyzed the impact of the alcohol intake status. This time, the grouping of former and current drinkers was successful to clearly exhibit significantly different outcomes as compared to nondrinkers. Indeed, in terms of OS, the rate of death due to cancer was significantly elevated in drinkers/former drinkers compared to nondrinkers (*p* = 0.003) (Fig. 4b).

We also analyzed the effect of the combination of active HPV infection and smoking status on OS (Fig. 4c). Globally, a comparison of 4 subgroups of patients did not reveal a significant difference between survival curves, but pairwise comparison demonstrated a significant difference in OS between HPV −/non-smokers and HPV −/smokers (*p = 0.025; HR 1.55; 95 % CI 1.06–2.27; Fig. 4c). The same comparison was carried out regarding drinking status, revealing a significant difference between the four curves (*p* = 0.02) (Fig. 4d). We proceeded to perform multiple pairwise comparisons and found that HPV− drinkers presented a poorer prognosis compared to HPV− nondrinkers (*p = 0.007; HR 2.04; 95 % CI 1.22–3.43; Fig. 4d). In this analysis, we also observed a tendency to a higher rate of death for active HPV+ drinker patients compared to HPV+ nondrinkers, but this association was not statistically significant because of the small group sizes (Fig. 4d). Finally, using the Fisher’s exact test, we examined the relation between HPV status and tobacco/alcohol status. The proportion of HPV+/p16+ patients was identical among non-smokers and smokers (Fig. 4e), whereas it was slightly higher among nondrinkers compared to drinkers. However, the latter difference was not significant (*p* = 0.1) (Fig. 4f).

### Multivariate analysis of HPV status and impact on prognosis

Multivariate Cox regression models detailed in Table 2 show that the HPV status has no independent prognostic value with regard to conventional risk factors and therapy response (which presented significant survival impacts in univariate analyses). In contrast, Table 2 reports significant prognostic values for stage (II/III vs. IV), alcohol as well as response to CCRT with regard to both PFS and OS (with an additional significant contribution of the smoking status to OS).

**Table 2:** Multivariate Cox regression model evaluating the impact of cancer staging, tobacco, alcohol consumption, HPV status and response to CCRT on progression-free survival (PFS), or overall survival (OS)

	Factors	HR	95 % CI	*p* value
PFS	Stage (II/III vs. IV)	1.78	1.17–2.71	<0.01
	Tobacco (non-smokers vs. smokers)	1.22	0.86–1.74	0.26
	Alcohol (nondrinkers vs. drinkers)	1.72	1.09–2.71	0.02
	HPV status (HPV− vs. active HPV+)	0.96	0.49–1.89	0.91
	Response to CCRT (no vs. yes)	3.38	2.40–4.78	<0.01
OS	Stage (II/III vs. IV)	1.60	0.99–2.59	0.05
	Tobacco (non-smokers vs. smokers)	1.51	1.03–2.22	0.03
	Alcohol (nondrinkers vs. drinkers)	2.27	1.34–3.86	<0.01
	HPV status (HPV− vs. active HPV+)	1.24	0.59–2.64	0.57
	Response to CCRT (no vs. yes)	2.93	2.01–4.29	<0.01

## Discussion

Locally advanced HPV+ HNSCCs represent a challenge for clinicians in terms of treatment strategy. This group of patients raises many therapeutic questions, including the choice of optimal treatment modality and the implications of HPV infection on the prognosis and response to CCRT. In our large population-based study, we demonstrated that the HPV status was neither associated with the response to CCRT nor the survival of HNSCC patients. We therefore reviewed previous studies examining HPV infection, response to CCRT and survival (Table 3). We noticed that very few studies have investigated correlations between such parameters and that they found a significant impact of HPV on the response to CCRT and an association with a better prognosis, unlike the findings reported in the current study (Kumar et al. 2007; Chung et al. 2009; Nichols et al. 2009; Fallai et al. 2009; de Jong et al. 2010; Ang et al. 2010; Rischin et al. 2010; Hong et al. 2010; Lill et al. 2011; Flavill et al. 2014; Hasegawa et al. 2014). This discrepancy with our findings can be explained by inclusion of smoker and/or drinker patients in our cohort and by the tumor location, which was not exclusively oropharyngeal. Moreover, smoking and drinking status was mostly imprecise or absent in previous studies, despite the fact that HPV+ tumors linked to tobacco and alcohol consumption represent a distinct biological and clinical entity. Indeed, Gillison and colleagues recently demonstrated that the outcome of treatment was compromised for p16+ and p16− patients who smoked during radiotherapy (Gillison et al. 2012). Unfortunately, HPV+ patients were rarely characterized according to the active nature of the infection, and even though an algorithm has been described that reliably identifies transcriptionally active HPV infection versus non-active infection in HNSCCs (Smeets et al. 2007). This distinction leads to a combination of p16 immunostaining followed by GP5 +/GP6 + PCR with 100 % specificity and sensibility. To our knowledge, ours is the first study to examine the implication of an active HPV infection in a large population of smoker/drinker HNSCCs and to reject the use of HPV as a predictive marker of response to treatment in this context.

**Table 3:** List of the studies reporting a positive impact of HPV on the response to chemoradiotherapy in HNSCCs

First author (year)	Number of patients	HPV prevalence (%)	Anatomical site	Smokers (*n*)	Drinkers (*n*)	Detection methods of HPV
Kumar et al. (2007)	42	64	Oropharynx	34	Not listed	qPCR
Chung et al. (2009)	46	50	Oropharynx	Not listed	Not listed	PCR In situ hybridization
Nichols et al. (2009)	44	61	Oropharynx	Not listed	Not listed	In situ hybridization
Fallai et al. (2009)	78	11	Oropharynx	Not listed	Not listed	qPCR
de Jong et al. (2010)	75	49	Pharynx Oral cavity	Not listed	Not listed	Genetic signature
Rischin et al. (2010)	172	65	Oropharynx	111	Not listed	PCR In situ hybridization
Hong et al. (2010)	35	24	Head and neck squamous cell carcinomas	Not listed	Not listed	qPCR
Ang et al. (2010)	323	64	Oropharynx	68	Not listed	In situ hybridization p16 immunohistochemistry
Lill et al. (2011)	29	38	Head and neck squamous cell carcinomas	Not listed	Not listed	PCR In situ hybridization
Hasegawa et al. (2014)	39	41	Oropharynx	16	33	PCR p16 immunohistochemistry
Flavill et al. (2014)	49	73	Oropharynx	28	12	PCR p16 immunohistochemistry

Wide geographic variation has been reported regarding tobacco and alcohol consumption in Europe. Indeed, in western and eastern countries, the vast majority of patients are avid consumers, whereas a greater decline in smoking habits was observed among Norwegian, Finnish, and Dutch populations (Giskes et al. 2005; Tinhofer et al. 2015). In this context, there remains a lack of studies assessing tobacco and alcohol exposure in HPV-driven versus tobacco- and alcohol-associated HNSCCs. Thus, considering our smoker/drinker population, we tried to clarify the impact of HPV infection on patient prognosis as well as that of classical risk factors. The major findings of our population-based study are that smoking and drinking significantly increased the rate of death within 5 years after diagnosis in head and neck cancer patients, and that the prognostic behavior of former smokers is similar to that of non-smokers, while that of former drinkers remains relatively poor, such as current drinkers. Our statistic-based observations are fully supported by clinical data reporting that clinical benefits are rapidly observed following the cessation of tobacco, whereas the adverse effects of alcohol impact the health over a longer term and are less easily reversible (Doll et al. 2004). Studies conducted in consumer patients with HNSCCs have already demonstrated the negative impact of smoking tobacco and drinking alcohol on treatment response and OS. Twenty years ago, Browman et al. first reported that patients who continue to smoke during radiation therapy have lower rates of response and survival than patients who do not smoke during radiation therapy (Browman et al. 1993). These results were consistent across many studies that have found that smoking and drinking behavior can predict the clinical outcome of HNSCC patients (Dikshit et al. 2005; Park et al. 2006; Hilgert et al. 2009; Duffy et al. 2009; Chen et al. 2011; Hoff et al. 2012; Sharp et al. 2014). Indeed, through a large meta-analysis, Bagnardi et al. recently confirmed the higher risk of oral and pharyngeal cancer development for heavy drinkers compared to nondrinkers: Alcohol consumers have a 5.13 times higher relative risk of developing this type of tumor (Bagnardi et al. 2015).

The effect of tobacco use on disease recurrence was also examined among patients with HPV-positive oropharyngeal carcinomas. The typically good prognosis of HPV+ oropharyngeal carcinomas was not observed in our at-risk population. In fact, the HPV+ smoker group exhibited an increased risk of recurrence and distant metastases as well as reduced survival compared with the HPV+ non-smoker group (Maxwell et al. 2010). Many additional studies have found that HPV+ smokers exhibit reduced survival compared with HPV+ non-smokers, given the increased risk of both local recurrence and distant metastases in HPV+ smokers (Hafkamp et al. 2008; Kumar et al. 2008; Tribius et al. 2012; Lin et al. 2013).

Moreover, there is increasing support that HPV has developed several mechanisms to escape from immune surveillance and to maintain infection. Additionally, the tobacco use is known to suppress immune function, thereby facilitating persistent infection. Thus, the immunosuppressive mechanisms of smoking may prevent the patient from activating immunologic responses to eradicate the viral infection (Arnson et al. 2010). In this context, we speculate that there is an additive effect of smoking/drinking habits and HPV infection that leads to poorer outcomes in HNSCC patients, possibly due to DNA breaks resulting from tobacco usage in human cells during the process of HPV genome integration, which occurs at fragile sites or “hot spots” of DNA breakage. This mechanism thereby increases the carcinogenic potential of HPV (Hu et al. 2015). These observations suggest that smoking/drinking behavior and an immunosuppressive status promote HPV infection and persistence, leading to poor patient prognosis. These findings highlight the need to evaluate the role of tobacco and alcohol in the natural history of oral HPV infection and the progression to malignancy.

At this time, our data have demonstrated that active HPV infection cannot be used as a prognostic tool in non-oropharyngeal cancer patients. Our analysis is subject to limitations related to the low available number of HPV+/p16+ specimens as supported by a recent meta-analysis demonstrating that transcriptionally active infection rates are generally low for oral cavity and larynx cancer with 16.3 and 8.6 %, respectively (Gama et al. 2016). Nevertheless, our data clearly underscore that smoking and drinking during therapy significantly worsens patient prognosis and increases the risk of recurrence. As previously recommended (Gritz et al. 2005), all future clinical trials should measure tobacco and alcohol exposure to evaluate their effects on disease control alongside determining HPV status. Moreover, our data suggest that heavy tobacco and alcohol consumers who respond to CCRT should remain under close clinical and radiological follow-up at the end of treatment for the early detection of recurrences independent of HPV status and that clinicians should warn patients and encourage them to halt their consumption to better manage this high-risk subpopulation.

## References

[CR1] Fact Sheets by Population. http://globocan.iarc.fr/Pages/fact_sheets_population.aspx. Accessed 25 Mar 2016

[CR2] Ang KK, Harris J, Wheeler R (2010). Human papillomavirus and survival of patients with oropharyngeal cancer. N Engl J Med.

[CR3] Arnson Y, Shoenfeld Y, Amital H (2010). Effects of tobacco smoke on immunity, inflammation and autoimmunity. J Autoimmun.

[CR4] Bagnardi V, Rota M, Botteri E (2015). Alcohol consumption and site-specific cancer risk: a comprehensive dose-response meta-analysis. Br J Cancer.

[CR5] Bonner JA, Harari PM, Giralt J (2006). Radiotherapy plus cetuximab for squamous-cell carcinoma of the head and neck. N Engl J Med.

[CR6] Browman GP, Wong G, Hodson I (1993). Influence of cigarette smoking on the efficacy of radiation therapy in head and neck cancer. N Engl J Med.

[CR7] Chen AM, Chen LM, Vaughan A (2011). Tobacco smoking during radiation therapy for head-and-neck cancer is associated with unfavorable outcome. Int J Radiat Oncol Biol Phys.

[CR8] Chung Y-L, Lee M-Y, Horng C-F (2009). Use of combined molecular biomarkers for prediction of clinical outcomes in locally advanced tonsillar cancers treated with chemoradiotherapy alone. Head Neck.

[CR9] D’Souza G, Kreimer AR, Viscidi R (2007). Case-control study of human papillomavirus and oropharyngeal cancer. N Engl J Med.

[CR10] de Jong MC, Pramana J, Knegjens JL (2010). HPV and high-risk gene expression profiles predict response to chemoradiotherapy in head and neck cancer, independent of clinical factors. Radiother Oncol J Eur Soc Ther Radiol Oncol.

[CR11] Depuydt CE, Benoy IH, Bailleul EJ (2006). Improved endocervical sampling and HPV viral load detection by Cervex-Brush Combi. Cytopathol Off J Br Soc Clin Cytol.

[CR12] Depuydt CE, Boulet GV, Horvath CJ (2007). Comparison of MY09/11 consensus PCR and type-specific PCRs in the detection of oncogenic HPV types. J Cell Mol Med.

[CR13] Dikshit RP, Boffetta P, Bouchardy C (2005). Lifestyle habits as prognostic factors in survival of laryngeal and hypopharyngeal cancer: a multicentric European study. Int J Cancer.

[CR14] Doll R, Peto R, Boreham J, Sutherland I (2004). Mortality in relation to smoking: 50 years’ observations on male British doctors. BMJ.

[CR15] Duffy SA, Ronis DL, McLean S (2009). Pretreatment health behaviors predict survival among patients with head and neck squamous cell carcinoma. J Clin Oncol Off J Am Soc Clin Oncol.

[CR16] Duray A, Descamps G, Decaestecker C (2012). Human papillomavirus DNA strongly correlates with a poorer prognosis in oral cavity carcinoma. The Laryngoscope.

[CR17] Duray A, Descamps G, Decaestecker C (2013). Human papillomavirus predicts the outcome following concomitant chemoradiotherapy in patients with head and neck squamous cell carcinomas. Oncol Rep.

[CR18] Fakhry C, Gillison ML (2006). Clinical implications of human papillomavirus in head and neck cancers. J Clin Oncol Off J Am Soc Clin Oncol.

[CR19] Fakhry C, Westra WH, Li S (2008). Improved survival of patients with human papillomavirus-positive head and neck squamous cell carcinoma in a prospective clinical trial. J Natl Cancer Inst.

[CR20] Fakhry C, Zhang Q, Nguyen-Tan PF (2014). Human papillomavirus and overall survival after progression of oropharyngeal squamous cell carcinoma. J Clin Oncol Off J Am Soc Clin Oncol.

[CR21] Fallai C, Perrone F, Licitra L (2009). Oropharyngeal squamous cell carcinoma treated with radiotherapy or radiochemotherapy: prognostic role of TP53 and HPV status. Int J Radiat Oncol Biol Phys.

[CR22] Flavill E, Fang YV, Miles B (2014). Induction chemotherapy followed by concurrent chemoradiotherapy for advanced stage oropharyngeal squamous cell carcinoma with HPV and P16 testing. Ann Otol Rhinol Laryngol.

[CR23] Forastiere AA, Goepfert H, Maor M (2003). Concurrent chemotherapy and radiotherapy for organ preservation in advanced laryngeal cancer. N Engl J Med.

[CR24] Gama RR, Carvalho AL, Filho AL (2016). Detection of human papillomavirus in laryngeal squamous cell carcinoma: systematic review and meta-analysis. Laryngoscope.

[CR25] Gillison ML, Zhang Q, Jordan R (2012). Tobacco smoking and increased risk of death and progression for patients with p16-positive and p16-negative oropharyngeal cancer. J Clin Oncol Off J Am Soc Clin Oncol.

[CR26] Giskes K, Kunst AE, Benach J (2005). Trends in smoking behaviour between 1985 and 2000 in nine European countries by education. J Epidemiol Community Health.

[CR27] Gritz ER, Dresler C, Sarna L (2005). Smoking, the missing drug interaction in clinical trials: ignoring the obvious. Cancer Epidemiol Biomark Prev Publ Am Assoc Cancer Res Cosponsored Am Soc Prev Oncol.

[CR28] Hafkamp HC, Manni JJ, Haesevoets A (2008). Marked differences in survival rate between smokers and nonsmokers with HPV 16-associated tonsillar carcinomas. Int J Cancer.

[CR29] Hasegawa M, Maeda H, Deng Z (2014). Prediction of concurrent chemoradiotherapy outcome in advanced oropharyngeal cancer. Int J Oncol.

[CR30] Hilgert E, Bergmann C, Fichtner A (2009). Tobacco abuse relates to significantly reduced survival of patients with oropharyngeal carcinomas. Eur J Cancer Prev Off J Eur Cancer Prev Organ ECP.

[CR31] Hoff CM, Grau C, Overgaard J (2012). Effect of smoking on oxygen delivery and outcome in patients treated with radiotherapy for head and neck squamous cell carcinoma–a prospective study. Radiother Oncol J Eur Soc Ther Radiol Oncol.

[CR32] Hong AM, Dobbins TA, Lee CS (2010). Human papillomavirus predicts outcome in oropharyngeal cancer in patients treated primarily with surgery or radiation therapy. Br J Cancer.

[CR33] Hu M, Ampil F, Clark C (2012). Comorbid predictors of poor response to chemoradiotherapy for laryngeal squamous cell carcinoma. The Laryngoscope.

[CR34] Hu Z, Zhu D, Wang W (2015). Genome-wide profiling of HPV integration in cervical cancer identifies clustered genomic hot spots and a potential microhomology-mediated integration mechanism. Nat Genet.

[CR35] Hutcheson KA, Lewin JS (2012). Functional outcomes after chemoradiotherapy of laryngeal and pharyngeal cancers. Curr Oncol Rep.

[CR36] Isayeva T, Li Y, Maswahu D, Brandwein-Gensler M (2012). Human papillomavirus in non-oropharyngeal head and neck cancers: a systematic literature review. Head Neck Pathol.

[CR37] Kumar B, Cordell KG, Lee JS (2007). Response to therapy and outcomes in oropharyngeal cancer are associated with biomarkers including human papillomavirus, epidermal growth factor receptor, gender, and smoking. Int J Radiat Oncol Biol Phys.

[CR38] Kumar B, Cordell KG, Lee JS (2008). EGFR, p16, HPV Titer, Bcl-xL and p53, sex, and smoking as indicators of response to therapy and survival in oropharyngeal cancer. J Clin Oncol Off J Am Soc Clin Oncol.

[CR39] Lazarus CL (2009). Effects of chemoradiotherapy on voice and swallowing. Curr Opin Otolaryngol Head Neck Surg.

[CR40] Lee L-A, Huang C-G, Liao C-T (2012). Human papillomavirus-16 infection in advanced oral cavity cancer patients is related to an increased risk of distant metastases and poor survival. PLoS ONE.

[CR41] Lill C, Kornek G, Bachtiary B (2011). Survival of patients with HPV-positive oropharyngeal cancer after radiochemotherapy is significantly enhanced. Wien Klin Wochenschr.

[CR42] Lin BM, Wang H, D’Souza G (2013). Long-term prognosis and risk factors among patients with HPV-associated oropharyngeal squamous cell carcinoma. Cancer.

[CR43] Maxwell JH, Kumar B, Feng FY (2010). Tobacco use in human papillomavirus-positive advanced oropharynx cancer patients related to increased risk of distant metastases and tumor recurrence. Clin Cancer Res Off J Am Assoc Cancer Res.

[CR44] Nichols AC, Faquin WC, Westra WH (2009). HPV-16 infection predicts treatment outcome in oropharyngeal squamous cell carcinoma. Otolaryngol-Head Neck Surg Off J Am Acad Otolaryngol-Head Neck Surg.

[CR45] Nygård M, Aagnes B, Bray F (2012). Population-based evidence of increased survival in human papillomavirus-related head and neck cancer. Eur J Cancer Oxf Engl.

[CR46] Park SM, Lim MK, Shin SA, Yun YH (2006). Impact of prediagnosis smoking, alcohol, obesity, and insulin resistance on survival in male cancer patients: National Health Insurance Corporation Study. J Clin Oncol Off J Am Soc Clin Oncol.

[CR47] Rischin D, Young RJ, Fisher R (2010). Prognostic significance of p16INK4A and human papillomavirus in patients with oropharyngeal cancer treated on TROG 02.02 phase III trial. J Clin Oncol Off J Am Soc Clin Oncol.

[CR48] Rosenquist K, Wennerberg J, Annertz K (2007). Recurrence in patients with oral and oropharyngeal squamous cell carcinoma: human papillomavirus and other risk factors. Acta Otolaryngol (Stockh).

[CR49] Rosenthal DI, Harari PM, Giralt J (2015). Association of Human Papillomavirus and p16 Status With Outcomes in the IMCL-9815 Phase III Registration Trial for Patients With Locoregionally Advanced Oropharyngeal Squamous Cell Carcinoma of the Head and Neck Treated With Radiotherapy With or Without Cetuximab. J Clin Oncol Off J Am Soc Clin Oncol.

[CR50] Sawicka M, Pawlikowski J, Wilson S (2013). The specificity and patterns of staining in human cells and tissues of p16INK4a antibodies demonstrate variant antigen binding. PLoS ONE.

[CR51] Sharp L, McDevitt J, Carsin A-E (2014). Smoking at diagnosis is an independent prognostic factor for cancer-specific survival in head and neck cancer: findings from a large, population-based study. Cancer Epidemiol Biomark Prev.

[CR52] Smeets SJ, Hesselink AT, Speel E-JM (2007). A novel algorithm for reliable detection of human papillomavirus in paraffin embedded head and neck cancer specimen. Int J Cancer.

[CR53] Sturgis EM, Cinciripini PM (2007). Trends in head and neck cancer incidence in relation to smoking prevalence: an emerging epidemic of human papillomavirus-associated cancers?. Cancer.

[CR54] Tinhofer I, Jöhrens K, Keilholz U (2015). Contribution of human papilloma virus to the incidence of squamous cell carcinoma of the head and neck in a European population with high smoking prevalence. Eur J Cancer Oxf Engl.

[CR55] Tribius S, Hoffmann AS, Bastrop S (2012). HPV status in patients with head and neck of carcinoma of unknown primary site: HPV, tobacco smoking, and outcome. Oral Oncol.

[CR56] Trotti A, Bellm LA, Epstein JB (2003). Mucositis incidence, severity and associated outcomes in patients with head and neck cancer receiving radiotherapy with or without chemotherapy: a systematic literature review. Radiother Oncol J Eur Soc Ther Radiol Oncol.

